# Grandparents Caring for Grandchildren, Family Structure, and Depressive Symptoms in China

**DOI:** 10.1093/geroni/igaa057.240

**Published:** 2020-12-16

**Authors:** Fengyan Tang, Ke Li

**Affiliations:** University of Pittsburgh, Pittsburgh, Pennsylvania, United States

## Abstract

It is a cultural norm for Chinese older adults to engage in co-parenting and caring for grandchildren. Previous research documented health advantages for grandparents who provide occasional, extensive, or even custodial care to grandchildren in China. Yet there is little information regarding the impacts of living arrangement and its interaction with grandchild care on grandparents’ psychological well-being. Using three waves of the 2011-2015 China Health and Retirement Longitudinal Study (CHARLS) data, this study examined the longitudinal association of depressive symptoms with grandchild care intensity and living arrangement among adults aged 40 and above (N=5,037). Mixed effects regression models were applied to examine changes in depressive symptoms and the associations with explanatory variables. At baseline, about half of respondents reported caring for their grandchild (ren). And nine percent lived with grandchildren only, that is, in a skipped-generation household and taking a custodial grandparent role. Overall, depressive symptoms did not change over time. After controlling for sociodemographic and health covariates, we found that providing medium level of care (i.e., between three to 10 hours per day) was associated with fewer baseline depressive symptoms, whereas grandparents living with grandchildren had more symptoms at baseline relative to those living with others. Further, an increased level of caregiving in the skipped-generation households was associated with more depressive symptoms. Given that custodial grandparenting is a growing phenomenon in China, further research needs to investigate how to reduce caregiving burden and associated adversary effects and how to promote overall well-being in this population.

